# Genome-wide association data classification and SNPs selection using two-stage quality-based Random Forests

**DOI:** 10.1186/1471-2164-16-S2-S5

**Published:** 2015-01-21

**Authors:** Thanh-Tung Nguyen, Joshua Zhexue Huang, Qingyao Wu, Thuy Thi Nguyen, Mark Junjie Li

**Affiliations:** 1 Shenzhen Key High Performance Data Mining Laboratory, Shenzhen Institutes of Advanced Technology, Chinese Academy of Sciences, 1068 Xueyuan Avenue, 518055 Shenzhen, China; 2 School of Computer Science and Engineering, Water Resources University, Hanoi, Vietnam; 3 University of Chinese Academy of Sciences, 100049 Beijing, China; 4 College of Computer Science and Software Engineering, Shenzhen University, Shenzhen, China; 5 School of Software Engineering, South China University of Technology, Guangzhou, China; 6 Faculty of Information Technology, Vietnam National University of Agriculture, Hanoi, Vietnam

**Keywords:** Genome-wide association study, SNPs Selection, Random Forests, Data mining

## Abstract

**Background:**

Single-nucleotide polymorphisms (SNPs) selection and identification are the most important tasks in Genome-wide association data analysis. The problem is difficult because genome-wide association data is very high dimensional and a large portion of SNPs in the data is irrelevant to the disease. Advanced machine learning methods have been successfully used in Genome-wide association studies (GWAS) for identification of genetic variants that have relatively big effects in some common, complex diseases. Among them, the most successful one is Random Forests (RF). Despite of performing well in terms of prediction accuracy in some data sets with moderate size, RF still suffers from working in GWAS for selecting informative SNPs and building accurate prediction models. In this paper, we propose to use a new two-stage quality-based sampling method in random forests, named ts-RF, for SNP subspace selection for GWAS. The method first applies *p*-value assessment to find a cut-off point that separates informative and irrelevant SNPs in two groups. The informative SNPs group is further divided into two sub-groups: highly informative and weak informative SNPs. When sampling the SNP subspace for building trees for the forest, only those SNPs from the two sub-groups are taken into account. The feature subspaces always contain highly informative SNPs when used to split a node at a tree.

**Results:**

This approach enables one to generate more accurate trees with a lower prediction error, meanwhile possibly avoiding overfitting. It allows one to detect interactions of multiple SNPs with the diseases, and to reduce the dimensionality and the amount of Genome-wide association data needed for learning the RF model. Extensive experiments on two genome-wide SNP data sets (Parkinson case-control data comprised of 408,803 SNPs and Alzheimer case-control data comprised of 380,157 SNPs) and 10 gene data sets have demonstrated that the proposed model significantly reduced prediction errors and outperformed most existing the-state-of-the-art random forests. The top 25 SNPs in Parkinson data set were identified by the proposed model including four interesting genes associated with neurological disorders.

**Conclusion:**

The presented approach has shown to be effective in selecting informative sub-groups of SNPs potentially associated with diseases that traditional statistical approaches might fail. The new RF works well for the data where the number of case-control objects is much smaller than the number of SNPs, which is a typical problem in gene data and GWAS. Experiment results demonstrated the effectiveness of the proposed RF model that outperformed the state-of-the-art RFs, including Breiman's RF, GRRF and wsRF methods.

## Background

The availability of high-throughput genotyping technologies has greatly advanced biomedical research, enabling us to detect genetic variations that are associated with the risk of diseases with much finer resolution than before. With genome-wide genotyping of single nucleotide polymorphisms (SNPs) in the human genome, it is possible to evaluate disease-associated SNPs for helping unravel the genetic basis of complex genetic diseases [1]. SNPs are single nucleotide variations of DNA base pairs, and it has been well established in the genome-wide association studies (GWAS) field that SNP profiles characterize a variety of diseases [2]. In light of emerging research on GWAS, hundreds or thousands of objects (with disease or normal controls) are collected, each object is genotyped at up to millions of SNPs. This is a typical problem of the number of SNPs is typically thousands of times larger than the number of objects. The task is to identify genetic susceptibility of SNPs through assaying and analyzing SNPs at the genome-wide scale [3].

A number of methods for analyzing of susceptibility of SNPs in GWAS have been proposed in the literature, where each SNP is analyzed individually [4]. However, it is found that only a small portion of the SNPs have main effects on the complex disease traits, but most of the SNPs have shown little penetrance individually. On the other hand, many common diseases in humans have been shown to be caused by complex interactions among multiple SNPs. This is known as multilocus interactions [5].

For dealing with the later challenge, one way of testing the interactions is to exhaustive search the interactions between all SNPs. The approach to test all two-SNPs to see how they are related to diseases is already quite time demanding [6]. Further exhaustive search for higher order interactions becomes computationally impractical because the number of tests increases exponentially with the order of interaction [7]. One approach to overcoming the drawbacks of the large computational cost using traditional statistical test is to first find a small set of high relevant SNPs using univariate tests on each SNP by discarding SNPs with high *p*-values, and then evaluate the interactions within the high relevant SNP subset [8].

This paper focuses on an approach for selecting informative SNPs, i.e. a small portion of the SNPs that has main effects on the disease, using Random Forests (RF) model [9]. RF has been applied successfully to genetic data in various studies [10-14]. A number of studies has used RFs to rank SNP predictors [15], to predict disease using SNPs [16] and to identify the effects related to diseases [17].

RF is an ensemble method for classification built from a set of decision trees that grow in randomly selected subspaces of data. Each tree is built using a bootstrap sample of objects. At each node, a random subspace of all SNPs is chosen to determine the best split to generate the children nodes. The size of subspace is referred to a parameter *mtry *that is used in growing the trees. Each node in a grown tree corresponds to a specific best predictor SNP in a subspace with *mtry *randomly selected SNPs. A grown tree in a forest is represented by a top-down decision tree, in which multiple decision paths from the root to different leaves go through the tree via various nodes. A decision path is a sequence of multiple SNPs including potential interactions between them in terms of hierarchial dependencies. In this way, RF can normally take into account interactions between SNPs (for details, see [18,19]).

A grown RF is able to yield a classification result and a measure of the feature importance for each SNP [18]. Although it is anticipated that RF will help to detect the SNP interactions, the task of selecting the relevant SNPs associated with complex disease in high dimensional genomewide data using RF method still poses significant challenges. In general, the SNP importance measure used to select the relevant SNPs is based on the impact of an SNP in predicting the response. The effectiveness of SNP importance depends on the performance of the grown RF that correctly classifies new objects of given SNPs.

A series of comprehensive studies revealed that the original RF implementation by Breiman is efficient to analyze low dimensional data. Bureau et al. [10] show that RF has worked well in a candidate gene case-control study involving only 42 SNPs. Lunetta et al. [19] show that RF can be applied to simulated data sets with no more than 1000 SNPs. However, it is computationally inefficient to build an accuracy RF model for high dimensional data. As a consequence, RF has rarely been applied on the genome-wide level for SNP selection and classification. Specifically, the original RF implementation designed to use a small default SNP subspace size *mtry*, e.g., *log*_2_*M *+ 1, is only suitable for low dimensional data, where *M *is the total number of SNPs. For high dimensional SNP data, there is usually a large number of SNPs that is considered to be irrelevant to the response, and only a small number of SNPs is relevant or informative. The simple random sampling method using a small *mtry *selects many subspaces without informative SNPs, and the number of objects is usually insufficient to generate numerous nodes to make it up to a good performance. To guarantee the performance of the generated RF model, previous studies recommended to use a relative large *mtry *in growing the trees of a RF when dealing with high dimensional data such as SNP data in GWA studies. However, the computational cost of the procedure of searching such a good *mtry *is very high which is dependent on the possible candidates to be searched. In the study by Schwarz et al. [20], a multiple sclerosis case control data set comprised of 325,807 SNPs in 3,362 individuals was used and it took 1 week to generate a full random forest on a server with 82 GHx CPU and 32 GB of memory, where the *mtry *values to search are $\sqrt{\;M}$, $2\sqrt{M}$, 0.1*M *, 0.5*M *and *M *. It was found that RFs built by small *mtry *values for high dimensional SNP data had poor classification performance [21].

In this paper, we propose to use a new approach in learning RFs model using a two-stage quality-based SNP subspace selection method, which is specifically tailored for high dimensional data of GWA studies. The proposed R-F model is computationally efficient to analyze GWA data sets with thousands to millions of SNPs without the need of using a large value of *mtry*. Furthermore, it is able to deliver a better classification performance than the original RF implementation using large *mtry *with a large margin. Our idea is to first add shadow SNPs into the original GWA data set. The shadow SNPs do not have prediction power to the outcome. However, they can give an indicator for the selection of informative SNPs. We then apply a *permutation procedure *to this extended GWA data to produce importance scores for all SNPs. The *p*-value assessment is used to find a cut-off point that separates informative SNPs from the noisy ones. Any SNP whose importance score is greater than the maximum importance score of the shadow SNPs is considered as important. We then use some statistical measures to split the set of informative SNPs into two groups: highly informative SNPs and weak informative SNPs. When sampling an SNP subspace for building trees, we only select SNPs from these two groups. This maintains the randomness of RFs meanwhile assuring the selection of informative SNPs. The resulting RF model is able to achieve a lower prediction error and avoid overfitting.

We conduct a series of experiments on two genome-wide SNP data sets (Parkinson disease case-control data set comprised of 408, 803 SNPs and Alzheimer case-control data set comprised of 380, 157 SNPs) to demonstrate the effectiveness of the proposed RF method. To validate the the conjecture that the approach is effective for problems with large *M *and small *N *, where *N *denotes the number of objects, we have conducted additional experiments on 10 other gene data sets with gene expression classification problems. Experimental results show that the proposed RF using two-stage quality-based SNP sampling can generate better random forests with higher accuracy and lower errors than other existing random forests methods, including Breiman's RF, GRRF and wsRF methods.

## Methods

Given a training data $L=\left\{{\left(X_{i},Y_{i}\right)}_{i=1}^{N}|X_{i}\in {\mathbb{R}}^{M},Y\in Y\right\}$, where *X_i _*are predictor SNPs, Y∈Y∈{1,2,…,c} is the outcome containing possible classes (diseases), *N *is the number of training samples (also called case-control objects) and *M *is the number of SNPs. Random Forests [9] independently and uniformly samples with replacement the training data  L to draw a bootstrap data set $L_{k}^{*}$ from which a decision tree $T_{k}^{*}$ is grown. Repeating this process for *K *replicates produces *K *bootstrap data sets and *K *corresponding decision trees $T_{1}^{*},T_{2}^{*},\ldots ,T_{k}^{*}$ which form a RF. Given an input *X *= *x*, let ${ĥ}_{k}\left(x\right)$ denote the prediction of class *j *of input $x\in {\mathbb{R}}^{M}$ by the *k*th tree, the RF prediction is obtained by aggregating the results given by all *K *decision trees, denoted as $\hat{Y}$, that is

(1) $$\hat{Y}=\text{arg}\underset{j\in Y}{\text{max}}\left\{\overset{K}{\underset{k=1}{\sum }}I\left[{ĥ}_{k}\left(x\right)=j\right]\right\},$$

where I(⋅) denotes the indicator function.

### Importance score of SNP from a RF

The importance score of SNPs can be obtained in growing trees [9]. At each node *t *in a decision tree, the split is determined by the decrease in node impurity. The node impurity is the gini index. If a sub-data set in node *t *contains samples from *c *classes (*c *≥ 2), the gini index is defined as $Gini\left(t\right)=1\sum_{j=1}^{c}{\hat{p}}_{j}^{2}$, where ${\hat{p}}_{j}^{2}$ is the relative frequency of class in *t*. *Gini*(*t*) is minimized if the classes in *t *are skewed. After splitting *t *into two child nodes *t*_1 _and *t*_2 _with sample sizes *N*_1_(*t*) and *N*_2_(*t*), the gini index of the split data is defined as

(2) $$Gini_{split}\left(t\right)=\frac{N_{1}\left(t\right)}{N\left(t\right)}Gini\left(t_{1}\right)+\frac{N_{2}\left(t\right)}{N\left(t\right)}Gini\left(t_{2}\right).$$

The SNP providing smallest *Gini_split_*(*t*) is chosen to split the node. The importance score of each SNP is computed over all *K *trees in a RF. These raw importance scores can be used to rank the SNPs.

### Two-stage quality-based SNP sampling method for subspace selection

The importance scores from a RF only give a simple ranking of SNPs. However, it is very difficult to select informative SNPs because of the noisy nature of the GWA data. For better subspace selection at each node of a tree, we first need to distinguish informative SNPs from noisy ones. Then, the informative SNPs are divided into two groups based on the statistical measures. When sampling the SNP subspace, SNPs from these groups are taken into account. Since the subspace always contains highly informative SNPs which can guarantee a better split at any node of a tree.

In the first stage we build *R *random forests to obtain raw importance scores and then use Wilcoxon rank-sum test [22] to compare the importance score of an SNP with the maximum importance scores of generated noisy SNPs called shadows. The shadow SNPs are added into the original GWA data set and they do not have prediction power to the outcome. From the replicates of shadow SNPs, we extracted the maximum value from each row of the importance score of the shadow SNP and put it into the comparison sample. For each SNP, we computed Wilcoxon test to check whether its mean importance score is greater than the maximum importance score of noisy SNPs. This test confirms that if a SNP is important, it consistently scores higher than the shadow over multiple permutations. Given a significance threshold *θ *(the default setting is 0.05), any SNP whose *p*-value is greater than *θ *is considered to be an irrelevant SNP and is removed from the system, otherwise, the relationship with the outcome is assessed. This method has been presented in [23].

In the second stage, we find the best subset of SNPs which is highly related to the outcome. We now only consider the subset of SNPs $\tilde{\text{X}}$ obtained from  L after neglecting all irrelevant SNPs and use a measure correlation function *χ*_2_($\tilde{\text{X}}$, *Y*) to test the association between the outcome label and each SNP *X_j_*. Let **X***_s _*be the best subset of SNPs, we collect all SNPs *X_j _*whose *p*-value is smaller than or equal to 0.05 as a result from the *χ*_2 _statistical test. The remaining SNPs {$\tilde{\text{X}}$\**X***s*} are added into **X***_w_*.

We independently sample SNPs from the two subsets and merge them together as the subspace SNPs for splitting the data at any node. The two subsets partition the set of informative SNPs in data without irrelevant SNPs. Given **X***_s _*and **X***_w_*, at each node, we randomly select *mtry *(*mtry *> 1) SNPs from each group of SNPs. For a given subspace size, we can choose proportions between highly informative SNPs and weak informative SNPs that depends on the size of the two groups. That is *mtrys *= [*mtry *× (||**X***_s_||/||*$\tilde{\text{X}}$||)] and *mtry_w _*= [*mtry *× (||**X***_w_||/||*$\tilde{\text{X}}$||)], where **X***_s _*and **X***_w _*are the number of SNPs in the groups of highly informative SNPs **X***_s _*and weak informative SNPs **X***_w_*, respectively. ||$\tilde{\text{X}}$|| is the number of informative SNPs in the input GWA data set. These are merged to form the SNP subspace for splitting the nodes in trees. This new sampling method always provides highly informative SNPs for the subspace at any node in growing a decision tree.

### The RF algorithm using two-stage quality-based SNP sampling method

We now present the random forest algorithm called ts-RF using a new SNP sampling method to generate splits at the nodes of CART trees [24]. The new algorithm is summarized as follows.

(1) Generate the extended data set of 2*M *dimensions by permuting the corresponding predictor SNP values for shadow SNPs.

(2) Build a random forest model *RF *from the extended data set and compute *R *replicates of raw importance scores of all SNPs and shadows with *RF *. Extract the maximum importance score of each replicate to form the comparison sample of *R *elements.

(3) For each SNP, take *R *importance scores and compute Wilcoxon test to get *p*-value.

(4) Given a significance level threshold *θ*, neglect all noisy SNPs.

(5) The *χ*^2 ^statistical test is used to separate the highly and weak informative subsets of SNPs **X***_s _*and **X***_w_*, respectively.

(6) Sample the training set  L with replacement to generate bagged samples $L_{k}$*, k *= 1, 2*, ..., K*.

(7) For each $L_{k}$, grow a CART tree *T_k _*as follows:

(a) At each node, select a subspace of *mtry *(*mtry *= *mtry_s _*+*mtry_w_, mtry >*1) SNPs randomly and separately from **X***_s _*and **X***_w _*and use the subspace SNPs as candidates for splitting the node.

(b) Each tree is grown nondeterministically, without pruning until the number of SNPs per leaf *n_min _*is reached.

(8) Given a *X *= *x_new_*, use Equation (1) to predict new samples on the test data set.

## Experiments

### Evaluation measures

We used Breiman's method as described in [9] to calculate the average *Strength *(*s*), the average *Correlation *(*ρ*) and *c/s*2 as performance measures of a random forest. Out-of-bag estimates were used to evaluate the strength and correlation. Given *s *and $\bar{\rho }$, the out-of bag estimate of the *c/s*2 measure can be computed with *ρ/s*^2^. The correlation measure indicates the independence of trees in a forest whereas the average strength correspond to the accuracy of individual trees. Low correlation and high average strength result in a reduction of general error bound measured by *c/s*2 which indicates a high accuracy RF model.

Let $D_{t}$ denote a test data set and *N_t _*denote the number of samples in $D_{t}$. The two measures are also used to evaluate the prediction performance of the RF models on $D_{t}$. One is the *Area under the curve *(AUC). The other one is the test accuracy, computed as:

(3) $$Acc=\frac{1}{N_{t}}\overset{N_{t}}{\underset{i=1}{\sum }}I\left(Q\left(x_{i},y_{i}\right)-max_{j\neq y_{i}}Q\left(x_{i},j\right)>0\right)$$

where *I*(·) is the indicator function, *y_i _*indicates the true class of $x_{i}\in D_{t}$ and $Q\left(x_{i},j\right)=\sum_{k=1}^{K}I\left({ĥ}_{k}\left(x_{k}\right)=j\right)$ the number of votes for *x_i _*on class *j*.

### Results on SNPs data sets

We conducted experiments on two genome-wide SNP data sets whose characteristics are summarized in Table 1 "Abbr" column indicates the abbreviation of the genome-wide SNP data sets used in the experiments.

**Table 1:** Description of two GWA data sets.

Data set	Abbr	#SNPs	#Cases-Controls	#Classes
Alzheimer	ALZ	380,157	364	2
Parkinson	PAR	408,803	541	2

The real data Alzheimer disease has been analyzed and reported in Webster et al. [25]. It contained genotypes of a total of 380,157 SNPs in 188 neurologically normal individuals (controls) and 176 Alzheimer disease patients (cases). The genotype data for Parkinson disease patients and controls were published in [26]. This genome-wide SNP consisted 271 controls and 270 patients with Parkinson disease, cerebrovascular disease, epilepsy, and amyotrophic lateral sclerosis. For raw genotype data with phs000089.v3.p2 study accession can be found in NCBI [1] dbGaP repository.

The 5-fold cross-validation was used to evaluate the prediction performance of the models on GWA data sets. From each fold, we built the models with 500 trees and the SNP partition was re-calculated on each training fold data set. We also compared the prediction performance of the ts-RF model with linear kernel SVM, taken from LibSVM [2], the values of regularization parameter by factors *C *were 2^-2 ^and 2^-5^, respectively. These optimal parameter *C *provided the highest validated accuracy on the training data set. The number of the minimum node size *n_min _*was 1. The parameters *R*, *mtry *and *θ *for pre-computation of the SNP partition were 30, 0.1*M *and 0.05, respectively. We used R to call the corresponding C/C++ functions from the ts-RF model and all experiments were conducted on the six 64bit Linux machines, each one equipped with Intel*R *Xeon*R *CPU E5620 2.40 GHz, 16 cores, 4 MB cache, and 32 GB main memory. The ts-RF and wsRF models were implemented as multi-thread processes, while other models were run as single-thread processes.

Table 2 shows the average of test accuracies and AUC of the models on the two GWA data asets using 5-fold cross-validation. We compare our ts-RF model with the Breiman's RF method and two recent proposed random forests models, that are the guided regularized random forests GRRF model [27] and the weighting subspace random forests wsRF model [28]. In the GRRF model, the weights are calculated using RF to produce importance scores from the out-of-bag data, in which these weights are used to guide the feature selection process. They found that the least regularized subset selected by their random forests with minimal regularization ensures better accuracy than the complete feature set. Xu et al. proposed a novel random forests wsRF model by weighting the input features and then selecting features to ensure that each subspace always contains informative features. Their efficient RF algorithm can be used to classify multi-class data.

**Table 2:** omparison of different random forests models on the SNP pair data sets with different *mtry *values.

Data set	Model	mtry setting	values	Acc	AUC
ALZ	ts-RF	$\sqrt{\;M}$	45	**.907**	**.975**
	wsRF	*log*_2_*M *	19	.561	.711
	wsRF	(*log*_2_*M*)^2^	361	.654	.729
	wsRF	$\sqrt{\;M}$	616	.692	.757
	GRRF	$\sqrt{\;M}$	616	.657	.706
	RF	*log*_2_*M*	19	.530	.623
	RF	$\sqrt{\;M}$	616	.632	.729
	RF	.1*M *	38,015	.654	.732
	RF	.5*M *	190,078	.663	.773
	SVM	C	2^−5^	.690	.716
PAR	ts-RF	$\sqrt{M_{p}}$	22	**.895**	**.959**
	wsRF	*log*_2_*M*	19	.754	.850
	wsRF	$\sqrt{\;M}$	638	.837	.917
	GRRF	$\sqrt{\;M}$	638	.688	.765
	RF	*log*_2_*M*	19	.564	.722
	RF	M	368	.799	.848
	RF	.1*M *	40,880	.808	.879
	RF	.5*M *	204,402	.827	.898
	SVM	C	2^−2^	.825	.902

Numbers in bold are the best results.

The latest RF [29] and GRRF [30] R-packages were used in R environment to conduct these experiments. For the GRRF model, we used a value of 0.1 for the coefficient *γ *because GRRF(0.1) has shown competitive prediction performance in [27]. We can see that ts-RF and wsRF always produced good results with a different *mtry *value. The ws-RF model achieved higher prediction accuracy when using $mtry=\sqrt{M}$. The ts-RF model using $mtry=\sqrt{M_{p}}$ outperformed the RF, GRRF, wsRF models and SVM on both GWA data sets, where *M_p _*= ||**X***_s_*|| + ||**X***_w_*|| denotes the number of informative SNPs. The RF model requires a larger number of SNPs to achieve better prediction accuracy (*mtry *= 0.5*M*). With this size, the computational time for building a random forest is still too high, especially for GWA data sets. It can be seen that the ts-RF model can select good SNPs in the subspace to achieve the best prediction performance. These empirical results indicate that, when classifying GWA data sets with ts-RF built from small yet informative subspaces, the achieved results can be satisfactory.

[1]http://www.ncbi.nlm.nih.gov/

[2]http://www.csie.ntu.edu.tw/˜cjlin/libsvmtools

Table 3 shows the prediction accuracy and Table 4 shows the *c/s*2 error bound of the random forest models with different numbers of trees while *mtry *= ⌊*log*_2_(*M *) + 1⌋ was fixed on the GWA data sets, respectively. We conducted these experiments to compare the new model with other random forests models and observed obvious improvement in classification accuracy on all GWA data sets. For the comparison of the *c/s*2 error bound, the GRRF model was not considered in this experiment because the RF model of Breimen's method [29] was used in the GRRF model as the classifier. The efficient wsRF model [28] and the Breimen's method were used for comparison in the experiment. We used the RF, wsRF and ts-RF models to generate random forests in different sizes from 20 trees to 200 trees and computed the average accuracy of the results from the 5-fold cross-validation. We can clearly see that the ts-RF model outperformed other models in classification accuracy and produced the lowest *c/s*2 error in most cases on all GWA data sets.

**Table 3:** The prediction test accuracy of the models on the SNP pair data sets against the number of trees *K*.

Data set	Model	K				
		
		20	50	80	100	200
ALZ	RF	.517	.491	.505	.555	.533
	GRRF	.503	.500	.539	.533	.528
	wsRF	.528	.588	.527	.602	.593
	ts-RF	**.711**	**.775**	**.791**	**.846**	**.893**
PAR	RF	.579	.557	.553	.597	.580
	GRRF	.532	.604	.641	.669	.680
	wsRF	.647	.680	.708	.710	.745
	ts-RF	**.852**	**.871**	**.858**	**.861**	**.871**

Numbers in bold are the best results.

**Table 4:** The (*c/s*2) error bound results of the models on the SNP pair data sets against the number of trees *K*.

Data set	Model	K				
		
		20	50	80	100	200
ALZ	RF	.2162	.1300	.0813	.0700	.0390
	wsRF	.2838	.1269	.0995	.1028	.0619
	ts-RF	**.1817**	**.0833**	**.0628**	**.0553**	**.0456**
PAR	RF	.2300	.1041	.0857	.0645	.0397
	wsRF	.2243	.1275	.0856	.0899	.0589
	ts-RF	**.1191**	**.0712**	**.0718**	**.0654**	**.0716**

Numbers in bold are the best results.

The proposed ts-RF model was applied to the Parkinson genome-wide data and assigned a score of importance to each SNP. The resulting list of SNPs was investigated for potential relevance to the Parkinson disease. Table 5 shows the results of the top 25 SNPs that are located within gene regions studied by the previous work. For each SNP, details including the rank value, SNP ID, gene symbol, gene ID, and p-value obtained using Wilcoxon test. The boldface rows in the table are the interesting genes associated with Parkinson disease. The results of this real data analysis validate the findings of GWA studies such as *PTPRD*, *EPHA4 *and *CAST*. Results also give other potential SNPs and genes that may be associated with the Parkinson disease. Specifically, some of these SNPs were found not to be strongly associated with the Parkinson disease by traditional statistical tests because they have relatively high p-value. This provides evidence of the advantages of using the proposed ts-RF model to detect potential SNPs associated with the disease. However, interpreting results and assessing their biological plausibility is challenging. Biologists can perform further investigation to validate their relationship with the Parkinson disease.

**Table 5:** Top 25 SNPs identified by ts-RF in Parkinson case-control data set.

Rank	SNP	Gene ID	Gene Symbol	p-value
1	rs7170952	64927	TTC23	2.1E-44
2	rs850084	101928208	LOC	3.6E-13
**3**	**rs832241**	**5789**	**PTPRD**	**6.8E-28**
4	rs1469593	647946	LINC00669	1.0E-34
5	rs9383311	9972	NUP153	1.5E-28
6	rs17023875	55591	VEZT	1.4E-32
7	rs9952724	9811	CTIF	3.7E-11
**8**	**rs3087584**	**2043**	**EPHA4**	**1.6E-04**
**9**	**rs10053056**	**831**	**CAST**	**6.4E-05**
10	rs6900852	135112	NCOA7	2.0E-08
11	rs3790577	10207	INADL	2.4E-25
12	rs722571	30000	TNPO2	1.6E-09
13	rs7924316	723961	INS-IGF2	1.1E-06
14	rs4956263	9811	CTIF	2.8E-06
15	rs12680546	165829	GPR156	1.3E-04
16	rs10518765	440279	UNC13C	1.5E-05
17	rs12185438	8715	NOL4	7.3E-05
18	rs12364577	440040	LOC440040	4.4E-10
19	rs2157787	463	ZFHX3	3.2E-03
20	rs17649	6692	SPINT1	1.0E-04
21	rs6429429	10000	AKT3	1.3E-03
**22**	**rs2346771**	**3084**	**NRG1**	**1.0E-02**
23	rs2666781	64215	DNAJC1	4.2E-04
24	rs2867301	55204	GOLPH3L	3.9E-03
25	rs11819434	282973	JAKMIP3	3.8E-02

Rows in bold indicate useful genes associated with neurological disorders.

In summary, ts-RF is a promising method for applying RF method to high-dimensional data such as GWA data. The application of ts-RF to GWA data may help to identify potential interesting SNPs that are difficult to be found with traditional statistical approaches.

### Results on gene data sets

To validate our conjecture that the proposed ts-RF model is effective for GWA data, we have conducted additional experiments on gene data sets. In this experiment, we compared across a wide range the performances of the 10 gene data sets, used in [31,27]. The characteristics of these data sets are given in Table 6. Using this type of data sets makes sense, since the number of genes of these data sets are much larger than the number of patients. For the RF method to obtain high accuracy, it is critical to select good genes that can capture the characteristics of the data and avoid overfitting at the same time.

**Table 6:** Description of 10 gene data sets.

Data set	**Abbr**.	#Genes	#Patients	#Classes
colon	COL	2,000	62	2
srbct	SRB	2,308	63	4
leukemia	LEU	3,051	38	2
lymphoma	LYM	4,026	62	3
breast.2.class	BR2	4,869	78	2
breast.3.class	BR3	4,869	96	3
nci 60	NCI	5,244	61	8
brain	BRA	5,597	42	5
prostate	PRO	6,033	102	2
adencarcinma	ADE	9,868	76	2

For the comparison of the models on gene data sets, we used the same settings as in [27]. For coefficient *γ *we used value of 0.1, because GR-RF(0.1) has shown a competitive accuracy [27] when applied to the 10 gene data sets. From each of gene data sets two-thirds of the data were randomly selected for training. The other one-third of the data set was used to validate the models. The 100 models were generated with different seeds from each training data set and each model contained 1000 trees. The *mtry *and *n_min _*parameters were set to $\sqrt{\;M}$ and 1, respectively. The prediction performances of the 100 classification random forest models were evaluated using Equation (3).

Table 7 shows the averages of the 100 repetitions of the *c/s*2 error bound when varying the number of genes per leaf *n_min_*. It can be seen that the RF, wsRF models produced lower error bound on the some data sets, for examples, COL, BR2, NCI and PRO. The ts-RF model produced the lowest *c/s*2 error bound on the remaining gene data sets on most cases. This implies that when the optimal parameters such as $mtry=\left[\sqrt{M}\right]$ and *n_min _*= 1 were used in growing trees, the number of genes in the subspace was not small and out-of-bag data was used in prediction, the results comparatively favored the ts-RF model. When the number of genes per leaf increased, so the depth of the trees was decreased, the ts-RF model obtained better results compared to other models on most cases, as shown in Table 7. These results demonstrated the reason that the two-stage quality-based feature sampling method for gene subspace selection can reduce the upper bound of the generalization error in random forests models.

**Table 7:** The (*c/s*2) error bound results of random forest models against the number of genes per leaf *nmin *on the ten gene data sets.

Data set	Model	*n_min_*					
		
		1	2	5	8	10	15
COL	RF	**.044**	**.032**	**.033**	**.032**	**.035**	**.034**
	wsRF	.046	.039	.042	.040	.042	.040
	ts-RF	.053	.043	.044	.043	.046	.044
SRB	RF	.018	.019	.017	.017	.019	.019
	wsRF	**.012**	.013	.013	.013	**.012**	.013
	ts-RF	.013	**.013**	**.011**	**.010**	.013	**.012**
LEU	RF	.040	.037	.037	.037	.039	.039
	wsRF	.035	.027	.028	.029	.032	.030
	ts-RF	**.023**	**.020**	**.021**	**.021**	**.022**	**.022**
LYM	RF	.019	.013	.012	.012	.016	.014
	wsRF	.011	.010	.010	.010	.010	.010
	ts-RF	**.008**	**.005**	**.005**	**.005**	**.007**	**.006**
BR2	RF	**.034**	**.034**	**.033**	**.035**	**.041**	**.037**
	wsRF	.038	.039	.038	.040	.042	.041
	ts-RF	.046	.039	.039	.039	.048	.045
BR3	RF	.068	**.056**	**.056**	**.054**	.064	**.057**
	wsRF	**.065**	.057	.057	.056	**.059**	.058
	ts-RF	.086	.064	.065	.062	.076	.066
NCI	RF	.037	.023	.024	.025	.030	.027
	wsRF	**.016**	**.017**	**.016**	**.017**	**.017**	**.017**
	ts-RF	.044	.022	.022	.025	.031	.025
BRA	RF	.045	.030	.029	.029	.028	.031
	wsRF	**.024**	.025	.024	.024	.024	.025
	ts-RF	.041	**.022**	**.022**	**.022**	**.021**	**.024**
PRO	RF	.041	.034	.033	.032	.037	.034
	wsRF	**.038**	.033	**.032**	**.031**	**.034**	**.032**
	ts-RF	.043	**.033**	.032	.032	.038	.033
ADE	RF	.080	.073	.071	.072	.076	.075
	wsRF	.068	.064	.065	.065	.065	.066
	ts-RF	**.054**	**.049**	**.048**	**.048**	**.051**	**.051**

Numbers in bold are the best results.

**Figure 1:**
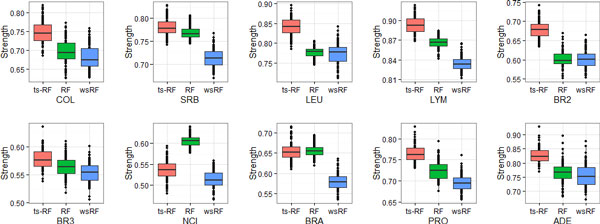
**Box plots of *Strength *measures on the 10 gene data sets with respect to *n_min _*= 1**.

**Figure 2:**
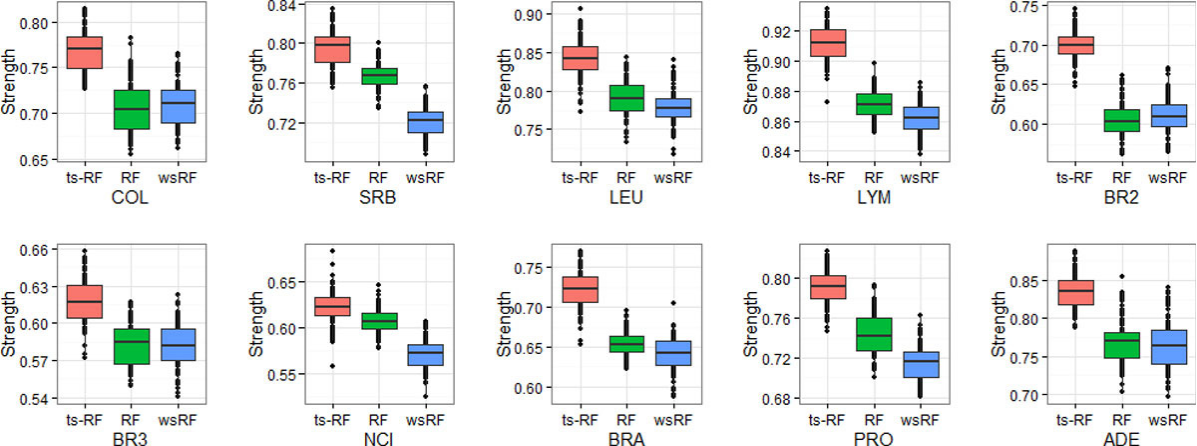
**Same as Figure 1, but for *n_min _*= 5**.

**Figure 3:**
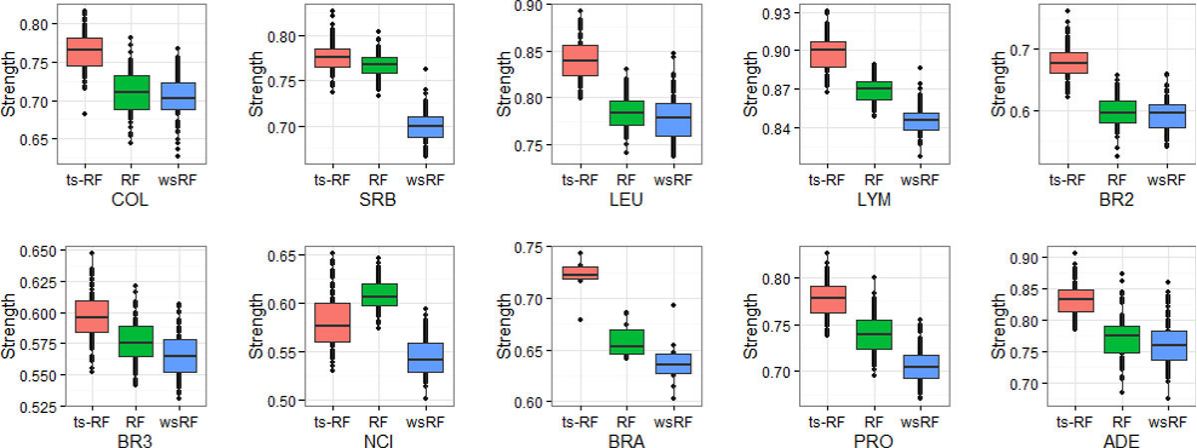
**Same as Figure 1, but for *n_min _*= 10**.

**Figure 4:**
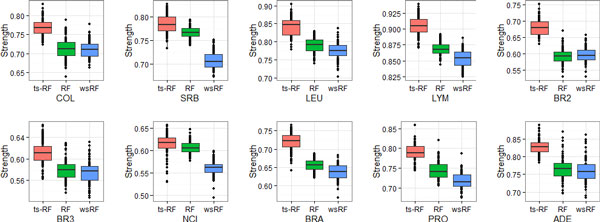
**Same as Figure 1, but for *n_min _*= 15**.

Figures 1, 2, 3, 4 show the effect of the two-stage quality-based feature sampling method on the strength measure of random forests. The 10 gene data sets were analyzed and results were compared to those of the random forests by Brieman's method and the wsRF model. In a random forest, the tree was grown from a bagging training data, the number of genes per leaf *n^min ^*varied from 1 to 15. Out-of bag estimates were used to evaluate the strength measure. From these figures, we can observe that the wsRF model obtained higher strength on the two data sets NCI and BRA when the number of genes per leaf was 1. The strength measure of the ts-RF model was the second rank on these two data sets and it was the first rank on the remaining gene data sets, as shown in Figure 1. Figures 2, 3, 4 demonstrate the effect of the depth of the tree, the ts-RF model provided the best results when varying the number of genes per leaf. This phenomenon implies that at lower levels of the tree, the gain is reduced because of the effect of splits on different genes at higher levels of the tree. The other random forests models reduce the strength measure dramatically while the ts-RF model always is stable and produces the best results. The effect of the new sampling method is clearly demonstrated in this result.

Table 8 shows the average test accuracy results (mean ± std-dev%) of the 100 random forest models computed according to Equation (3) on the gene data sets. The average number of genes selected by the ts-RF model, from 100 repetitions for each data set, are shown on the right of Table 8, divided into a strong group **X***_s _*and a weak group **X***_w_*. These genes were used by the two-stage quality-based feature sampling method in growing trees in ts-RF.

**Table 8:** Test accuracy results (mean ± std-dev%) of random forest models against the number of genes per leaf *n_min _*on the ten gene data sets.

Data set	Model	1 genes	2 genes	5 genes	8 genes	10 genes	15 genes	**X***_s_*	**X***_w_*
COL	RF	.844 ± 0.5	.818 ± 0.8	.832 ± 0.7	.830 ± 0.6	.849 ± 0.3	.853 ± 0.4		
	GRRF	.865 ± 0.5	.832 ± 0.6	.848 ± 0.5	.838 ± 0.6	.853 ± 0.3	.859 ± 0.3		
	wsRF	.845 ± 0.5	.837 ± 0.4	.857 ± 0.5	.834 ± 0.6	.844 ± 0.4	.848 ± 0.5		
	ts-RF	**.877 ± 0.4**	**.863 ± 0.4**	**.879 ± 0.3**	**.863 ± 0.5**	**.874 ± 0.3**	**.874 ± 0.3**	245	317
SRB	RF	.959 ± 0.3	.957 ± 0.2	.961 ± 0.2	.944 ± 0.5	.914 ± 1.0	.777 ± 1.2		
	GRRF	.976 ± 0.2	.972 ± 0.1	.972 ± 0.2	.941 ± 0.7	.898 ± 1.1	.802 ± 1.1		
	wsRF	.968 ± 0.3	.967 ± 0.3	.966 ± 0.3	.957 ± 0.3	.912 ± 0.5	.771 ± 0.2		
	ts-RF	**.977 ± 0.2**	**.974 ± 0.1**	**.977 ± 0.1**	**.962 ± 0.4**	**.922 ± 1.1**	**.812 ± 1.1**	606	546
LEU	RF	.826 ± 1.2	.849 ± 0.9	.866 ± 0.9	.879 ± 0.9	.871 ± 1.0	.874 ± 1.0		
	GRRF	.873 ± 0.9	.867 ± 0.7	.880 ± 0.9	.878 ± 0.9	.876 ± 0.9	.885 ± 0.9		
	wsRF	.848 ± 1.0	.848 ± 0.9	.863 ± 1.0	.858 ± 1.1	.851 ± 1.0	.866 ± 1.1		
	ts-RF	**.893 ± 0.7**	**.885 ± 0.6**	**.906 ± 0.7**	**.908 ± 0.7**	**.913 ± 0.7**	**.905 ± 0.7**	502	200
LYM	RF	.972 ± 0.2	.983 ± 0.1	.979 ± 0.3	.930 ± 1.1	.855 ± 1.2	.823 ± 0.6		
	GRRF	.991 ± 0.1	.989 ± 0.1	.983 ± 0.3	.928 ± 1.1	.840 ± 1.1	.805 ± 0.4		
	wsRF	.981 ± 0.2	.982 ± 0.2	.975 ± 0.4	.928 ± 0.2	.845 ± 0.3	.801 ± 0.2		
	ts-RF	**.993 ± 0.1**	**.995 ± 0.0**	**.987 ± 0.3**	**.935 ± 1.1**	**.856 ± 1.2**	**.828 ± 0.7**	1404	275
BR2	RF	.627 ± 0.7	.618 ± 0.7	.608 ± 0.7	.622 ± 0.7	.601 ± 0.7	.640 ± 0.7		
	GRRF	.713 ± 0.9	.623 ± 0.8	.615 ± 0.8	.627 ± 0.7	.617 ± 0.8	.643 ± 0.7		
	wsRF	.634 ± 0.7	.627 ± 0.8	.618 ± 0.8	.619 ± 0.9	.604 ± 0.8	.626 ± 0.7		
	ts-RF	**.788 ± 0.7**	**.766 ± 0.8**	**.776 ± 0.9**	**.776 ± 0.8**	**.765 ± 1.1**	**.780 ± 0.8**	194	631
BR3	RF	.560 ± 0.7	.568 ± 0.7	.560 ± 0.7	.581 ± 0.6	.563 ± 0.8	.567 ± 0.8		
	GRRF	.635 ± 0.8	.580 ± 0.6	.574 ± 0.7	.586 ± 0.6	.568 ± 0.7	.580 ± 0.8		
	wsRF	.572 ± 0.7	.575 ± 0.7	.571 ± 0.7	.579 ± 0.4	.565 ± 0.8	.580 ± 0.6		
	ts-RF	**.654 ± 0.7**	**.657 ± 0.7**	**.661 ± 0.6**	**.670 ± 0.6**	**.645 ± 0.7**	**.648 ± 0.9**	724	533
NCI	RF	.589 ± 1.1	.584 ± 1.3	.558 ± 1.2	.470 ± 1.2	.379 ± 1.5	.206 ± 0.9		
	GRRF	.631 ± 1.3	.592 ± 1.3	.561 ± 1.2	.483 ± 1.2	.403 ± 1.5	.228 ± 1.0		
	wsRF	.594 ± 1.1	.589 ± 1.4	.578 ± 1.0	.478 ± 1.2	.390 ± 1.5	.239 ± 1.4		
	ts-RF	**.742 ± 1.2**	**.731 ± 1.8**	**.684 ± 1.3**	**.552 ± 1.9**	**.430 ± 1.7**	**.248 ± 1.1**	247	1345
BRA	RF	.708 ± 1.6	.706 ± 2.0	.701 ± 1.7	.637 ± 2.1	.600 ± 3.0	.368 ± 3.0		
	GRRF	.748 ± 1.7	.729 ± 1.9	.726 ± 1.8	.654 ± 2.3	.650 ± 4.0	.416 ± 2.9		
	wsRF	.708 ± 1.8	.718 ± 1.9	.691 ± 1.8	.652 ± 1.7	.650 ± 3.2	.431 ± 2.3		
	ts-RF	**.819 ± 1.6**	**.815 ± 2.0**	**.783 ± 1.8**	**.694 ± 2.1**	**.679 ± 3.0**	**.405 ± 3.4**	1270	1219
PRO	RF	.887 ± 0.4	.894 ± 0.4	.895 ± 0.4	.891 ± 0.3	.882 ± 0.3	.891 ± 0.3		
	GRRF	.929 ± 0.2	.916 ± 0.2	.916 ± 0.2	.908 ± 0.3	.907 ± 0.3	.917 ± 0.2		
	wsRF	.908 ± 0.2	.911 ± 0.3	.913 ± 0.3	.906 ± 0.3	.897 ± 0.3	.908 ± 0.3		
	ts-RF	**.926 ± 0.2**	**.928 ± 0.1**	**.927 ± 0.2**	**.919 ± 0.2**	**.915 ± 0.2**	**.926 ± 0.2**	601	323
ADE	RF	.840 ± 0.4	.846 ± 0.4	.849 ± 0.3	.845 ± 0.4	.832 ± 0.3	.839 ± 0.3		
	GRRF	.855 ± 0.5	.842 ± 0.4	.848 ± 0.3	.848 ± 0.4	.832 ± 0.3	.834 ± 0.4		
	wsRF	.841 ± 0.4	.841 ± 0.4	.845 ± 0.3	.842 ± 0.4	.828 ± 0.3	.832 ± 0.3		
	ts-RF	**.909 ± 0.4**	**.906 ± 0.4**	**.904 ± 0.4**	**.902 ± 0.5**	**.888 ± 0.4**	**.901 ± 0.4**	108	669

Numbers in bold are the best results.

The results from the application of GRRF on the ten gene data sets were presented in [27]. From these prediction accuracy results in Table 8, the GRRF model provided slightly better result on SRB data set in case *n_min _*= 15 and PRO in case *n_min _*= 1, respectively. The wsRF model presented the best result on BRA and NCI data sets in case *n_min _*= 15. In the remaining cases on all gene data sets, the ts-RF model shows the best results. In some cases where ts-RF did not obtain the best results, the differences from the best results were minor. This was because the two-stage quality-based feature sampling was used in generating trees in the ts-RF, the gene subspace provided enough highly informative genes at any levels of the decision tree. The effect of the two-stage quality-based feature sampling is clearly demonstrated in these results.

## Conclusion

We have presented a two-stage quality-based random forests for genome-wide association data classification and SNPs selection. The presented approach has shown to be effective in selecting informative sub-groups of SNPs and potentially associated with diseases that traditional statistical approach might fail. The proposed random forests model works well for the data where the number of case-control objects is much smaller than the number of SNPs, which is a typical problem in GWAS.

We have conducted a series of experiments on the two genome-wide SNP and ten gene data sets to demonstrate the effectiveness of the proposed RF model. The top 25 SNPs in Parkinson data set were identified by the proposed RF model including some interesting genes associated with neurological disorders. Experimental results have shown the improvement in increasing test accuracy for GWA classification problems and reduction of the *c/s*2 error in comparison with other state-of-the-art random forests, including Breiman's RF, GRRF and wsRF methods.

## Competing interests

The authors declare that they have no competing interests.

## Authors' contributions

T. -T. Nguyen, J. Z. Huang and T.T. Nguyen participated in designing the algorithm, T. -T. Nguyen and Q. Wu drafted the manuscript. T. -T. Nguyen performed the implementations. J. Z. Huang revised and finalized the paper. All authors read and approved the final manuscript.

## Declarations

Publication of this article was funded by NSFC under Grant NO.61203294 and Natural Science Foundation of SZU under Grant NO.201433.

This article has been published as part of *BMC Genomics *Volume 16 Supplement 2, 2015: Selected articles from the Thirteenth Asia Pacific Bioinformatics Conference (APBC 2015): Genomics. The full contents of the supplement are available online at http://www.biomedcentral.com/bmcgenomics/supplements/16/S2
